# Predictors of compliance with community-directed treatment with ivermectin for onchocerciasis control in Kabo area, southwestern Ethiopia

**DOI:** 10.1186/s13071-015-0695-7

**Published:** 2015-02-15

**Authors:** Adugna Endale, Berhanu Erko, Fitsum Weldegebreal, Mengistu Legesse

**Affiliations:** DireDawa University, School of Medicine, P. O. Box 1362, Dire Dawa, Ethiopia; Aklilu Lemma Institute of Pathobiology, Addis Ababa University, P. O. Box 1176, Addis Ababa, Ethiopia; Haramaya University, College of Health and Medical Sciences, Department of Medical Laboratory Science, P. O. Box 235, Harar, Ethiopia

**Keywords:** Onchocerciasis, Ivermectin, Treatment, Compliance, Ethiopia

## Abstract

**Background:**

Compliance with annual ivermectin treatment is a major challenge in community-directed treatment with ivermectin (CDTI) implementation. There are individuals who do not comply with the annual mass treatment, which contributes to the continuity for disease transmission. Hence, ensuring high treatment coverage and sustained compliance should be given due emphasis in the control of onchocerciasis. The aim of this study was to determine CDTI compliance rate and predictors of compliance where the CDTI was in its 9^th^ round in Kabo area, southwestern Ethiopia.

**Methods:**

Community-based cross-sectional study was conducted in Kabo area, three weeks after the 9th round of annual ivermectin distribution. Systematic random sampling was used to select head of households and structured, pre-tested questionnaire was used to interview the study participants. Data was analyzed using SPSS version 16. Descriptive statistics was used to compute mean and standard deviation of continuous variables and frequency for categorical variables, while bivariate and multivariate logistic regressions were used to assess the effects of independent variables on the outcome variable. Variables which showed association in multivariate analysis were considered as final predictors of compliance and strength of association was measured through adjusted odds ratio (AOR).

**Results:**

A total of 308 respondents (age range 18-70, mean age ± SD, 32.21 ± 9.64) participated in the study. Of these, 249 (80.8%) reported that they took ivermectin during the 9^th^ round annual treatment. Significantly higher rate of treatment compliance was reported by participants age ≥35 years (AOR = 5.48, 95% CI; 1.97 - 15.23), participants who stayed in the area for more than ten years (AOR = 3.86, 95% CI; 1.83- 8.11), participants who perceive that they are at risk of contracting the disease(AOR = 7.05, 2.70- 18.43), participants who perceive community drug distributors (CDDs) are doing their work well (AOR = 2.35 95% CI; 1.15- 4.83) and participants who know at least one CDD in their village (AOR = 2.83, 95% CI; 1.26- 6.40).

**Conclusion:**

The majority of the study participants in the present study area complied with ivermectin treatment. Nevertheless, intervention packages should consider factors such as age, residence duration and community’s perception of the disease to improve compliance and make drug distribution sustainable.

## Background

Onchocerciasis is a debilitating vector-borne disease caused by the parasite, *Onchocerca volvulus*; transmitted by the bite of black-fly, *Simulium damnosum* [1]. The disease is public health and socio-economic threat in many African countries [2,3]. Worldwide there are more than 120 million people at risk of contracting the disease, while 18 million people are already infected. More than 99% of the disease burden is in Africa [4]. Onchocerciasis affects the working age population, and it is the second-leading infectious cause of blindness worldwide, being responsible for about 500,000 blindness [5,6].

In Ethiopia, it is estimated that 3 million people are already infected, whereas 7.3 million are at risk of infection. Nine regions surveyed for onchocerciasis were found to be endemic and the endemic areas extend from the northwest part to southwest part of the country [3,7,8]. Onchocerciasis has been targeted for control and eventually elimination, as a disease of public health and socio-economic problems in Ethiopia along with 19 other African countries [1].

Annual mass treatment with an oral microfilaricidal (ivermectin) is the main control strategy for onchocerciasis in Ethiopia [9]. A community-directed treatment with ivermectin (CDTI) strategy was adopted in line with the African Program for Onchocerciasis Control (APOC) since 1996. CDTI is a cost-effective strategy [6]. Several studies have shown that mass treatment with ivermectin is safe and effective in reducing the transmission of the disease [2,10-14]. The annual mass treatment must continue for about 15 years [15,16], while all eligible members of the community should take the drug in order to ensure an adequate reduction in the transmission of the parasite and protection from further infection [17]. Studies show that not all eligible members of the community receive the annual treatment [18]. Hence, compliance with annual ivermectin treatment has become a major challenge to put CDTI implementation on the ground.

Studies done elsewhere have shown that compliance with ivermectin treatment can be influenced by factors such as age and gender [16,19-21], beliefs about individual susceptibility to onchocerciasis [16,22], programme organization, perceived social influence and support, perceived benefits of ivermectin treatment [22], characteristics of the client, perceived performance of CDDs/providers [16,22] and methods of dose determination [16]. CDTI is currently on-going in Ethiopia. However, there is limited information regarding treatment compliance rate and factors affecting treatment compliance. Therefore, the aim of this study was to determine the rate of compliance with CDTI and identify factors influencing compliance with CDTI in Kabo area, which would contribute to the success of the on-going program.

## Methods

### Study area and population

Community-based cross-sectional study was conducted in Kabo area, Gambella Regional State, southwestern Ethiopia, between November and December 2012. The study area is located about 667 kilometers south west of Addis Ababa and 340 kilometers from Gambella Town. The major ethnic composition of the area include Amhara, Oromia, Tigre, Kafficho, Kambata, Shakacho, Mezhenger and some others from southern Ethiopia. Most of the inhabitants are engaged in mixed farming like coffee plantation, maize cultivation and rearing livestock. Based on the 2007 Ethiopian National Population and Housing Census, the population of the area is projected to be about 46,583, with 23,429 males and 23,154 females [23].

Community-based treatment with ivermectin (Mectizan®) for the control of onchocerciasis was initiated in the area in 2004 by World Health Organization (WHO)/APOC in partnership with Ethiopian Federal Ministry of Health (FMOH), The Carter Centre, the local administration and the communities [23]. Since then, ivermectin has been annually distributed to all eligible members of the community in the villages through CDDs [23], using the height and physical appearance dosing method [24]. According to the information obtained from Gambella Regional Health Bureau (GRHB), in 2011 the annual treatment coverage of ivermectin in the area was around 76% [23].

### Sample size estimation and data collection

To determine the number of participants to be included in the study, single population proportion formula was used with the assumption that: the proportion of ivermectin treated individuals (treatment compliers) in the community was 75.8% [23], confidence level 95% and degree of precision 5%. To compensate for the non-respondents, 10% was considered and thus, the final sample size was estimated to be 308.

The study area was stratified into four manageable villages based on their proximity. Then, the sample size was distributed proportionally to each village based on their population size. Systematic random sampling technique was used to recruit the households for this study, and heads of households (i.e. husband or wife or any representative), aged 18 years and older, and who stayed in the area for more than one year, apparently healthy, and volunteered to participate were included in this study. Pregnant and lactating women having infants younger than one month of age (during the data collection period) and individuals who were seriously sick were excluded from the study since they had already been excluded from the mass treatment.

A structured, pre-tested questionnaire was used for data collection. The questionnaire was adopted from similar studies conducted previously [16,22]. It has three major components: socio-demographic factors, behavioral factors (such as knowledge, belief and attitude/perception towards the treatment, the disease, and CDDs) and service related factors. The service related factors here refer to activities related to service provision such as timing of service provision (week end, working days, holidays), decision maker of ivermectin distribution (Woreda/District Office, CDDs, Community leaders, HEWs), places of service provision (home to home, fixed village centers and others) and sources of information about the service (family, local leaders, health workers, health extension workers, CDDs, friends, religious leaders, radio and others). The questionnaire was prepared in English and then, translated into Amharic and back to English to check for its consistency. The survey was conducted three weeks after the 9th round of annual ivermectin distribution in the area. The participants were interviewed in local languages by trained data collectors who were selected from the area and the interview made by house-to-house visit.

### Data entry and analysis

After the completion of data collection, cleaning, editing and coding was done. Then the data were entered using EpiData software, V.3.1 and analyzed using SPSS version 16.0. Descriptive statistics was used to compute mean and standard deviation of continuous variables and frequency for categorical variables. Bivariate and multivariate logistic regressions were used to assess the effects of independent variables on the outcome variable, while simultaneously controlling for other potential confounding factors. To identify independent predictors of compliance with ivermectin treatment, backward multivariate logistic regression method was used. Variables which showed association in multivariate analysis at 5% level of significance were considered as final predictors of compliance with ivermectin treatment. The strength of association between different exposure variables and the outcome variable was measured through adjusted odds ratio.

### Ethical considerations

The study was carried out after obtaining ethical approval from the Institutional Review Board (IRB) of Aklilu Lemma Institute of Pathobiology, Addis Ababa University. Then, permission was obtained from GRHB, Zonal Health Departments & District Health Offices. Before the interview commenced, informed verbal consent was obtained from all study participants after explaining the objective of the study to the participants.

## Results

### Socio-demographic characteristics of respondents

A total of 308 participants (age range 18-70 years, mean age 32.21 ± 9.64 years) were interviewed yielding a response rate of 100%. The mean length of stay of respondents in the study area was 17.13 ± 8.64 years. Table 1 shows the socio-demographic characteristics of the participants.

**Table 1:** **Socio-demographic characteristics of 308 study participants recruited from the community, Kabo area, southwestern Ethiopia, 2012**

**Variables**	**Category**	**Freq (%)**
**Sex**	Male	183 (59.4)
	Female	125 (40.6)
**Age group**	≤35	213 (69.2)
	>35	95 (30.8)
**Ethnicity**	Amhara	120 (39.0)
	Oromo	89 (28.9)
	Keffa	34 (11.0)
	Sheka	28 (9.1)
	Mezhenger	13 (4.2)
	Tigre	13 (4.2)
	Others	11 (3.6)
**Religion**	Orthodox	147 (47.7)
	Muslim	75 (24.4)
	Protestant	72 (23.4)
	Others	14 (4.5)
**Marital status**	Married	231 (75.0)
	Single	77 (25.0)
**Educational status**	Illiterate	144 (46.8)
	Literate	164 (53.2)
**Length of stay**	<10 Years	74 (24.0)
	≥10 Years	234 (76.0)
**Occupation**	Farmer	221 (71.8)
	Others	87 (28.2)

### CDTI compliance rate

About 80.8% (249/308) of the respondents took the treatment. The remaining 19.2% (36 males and 23 females) eligible individuals did not comply with the annual treatment. The mean age (±SD) of respondents who comply with the treatment and those that did not comply with the treatment was 32.92 (±9.35) and 29.20 (±1.03), respectively. The most frequently mentioned reasons for missing the treatment were: absence (due to farming) during the campaign day (37.3%), CDDs did not come to their house to provide them with the treatment (20.4%), do not trust the importance of the treatment (13.6%) and other reasons such as fear of side effects and shortage of drug (11.8%) (Table 2).

**Table 2:** **CDTI compliance rate of the study participants in Kabo area, southwestern Ethiopia, 2012**

**Variables (N = 308)**	**Category**	**Freq (%)**
**Did you receive ivermectin in the recent campaign (this year)?**	Yes	249 (80.8)
	No	59 (19.2)
**If no what was the reason? (n = 59)**	Absence (farming)	22 (37.3)
	CDDs not coming to their house	12 (20.4)
	Not being informed	10 (16.9)
	Believing free things are useless for health	8 (13.6)
	Others (fear of side effects, shortage of drug etc)	7 (11.8)
**From where do you collect the tablet? (n = 249)**	CDDs come to my house	213 (85.5)
	From village center	35 (14.1)
	From CDDs house	1 (0.4 )

### CDTI compliance rate versus socio-demographic factors

As shown in Table 3, the magnitude of compliance with ivermectin treatment did not show a significant difference within sex (80.3% for males and 81.6% for females) and marital status (81.0% for married and 80.5% for single respondents) categories (P > 0.05). Relatively, higher rate of treatment compliance was reported among respondents with age greater than 35 years (93.7%) compared to those with age 35 years and less (75.1%) (P < 0.05). Statistically significant difference was also observed with respect to length of stay. Compliance to treatment was higher for those who stayed in the area for ten years and more (87.2%) compared to those who stayed for less than ten years (60.8%) (P < 0.05). Educational status and ethnicity also showed a significant association with treatment compliance. Higher treatment compliance rate was reported by literate (86.0%) compared to illiterate (75.0%). Regarding family size, the magnitude of compliance to treatment seems higher for those with family size 1-4 (82.1%) compared to those of households having nine and more family members (68.2%), but it did not show a statistically significant difference (p > 0.05). There was no significant difference observed in compliance rate with regard to the type of occupation, 79.6% for farmers compared to 83.9% for those of respondents engaged in other occupations (p > 0.05).

**Table 3:** **CDTI compliance rate versus socio-demographic factors among the study participants in Kabo area, southwestern Ethiopia, 2012**

**Variables (N = 308)**	**Category (no.)**	**Compliance to Ivermectin**		**P-value**
		**Yes (%)**	**No (%)**	
**Sex**	Male (183)	147 (80.3)	36 (19.7)	0.78
	Female (125)	102 (81.6)	23 (18.4)	
**Age group**	≤35 Years (213)	160 (75.1)	53 (24.9)	0.00
	>35 Years (95)	89 (93.7)	6 (6.3)	
**Ethnicity**	Indigenous (13)	7 (53.8)	6 (46.2)	0.01
	Others (295)	242 (82.0)	53 (18.0)	
**Religion**	Orthodox (147)	122 (83.0)	25 (17.0)	0.20
	Muslim (75)	63 (84.0)	12 (16.0)	
	Protestant (72)	52 (72.2)	20 (27.8)	
	Others (14)	12 (85.7)	2 (14.3)	
**Marital status**	Married (231)	187 (81.0)	44 (19.0)	0.93
	Single (77)	62 (80.5)	15 (19.5)	
**Educational status**	Illiterate (144)	108 (75.0)	36 (25.0)	0.02
	Literate (164)	141 (86)	23 (14)	
**Family size**	1-4 (173)	142 (82.1)	31 (17.9)	0.29
	5-8 (113)	92 (81.4)	21 (18.6)	
	9^+^ (22)	15 (68.2)	7 (31.8)	
**Length of stay**	<10 Years (74)	45 (60.8)	29 (39.2)	0.00
	≥10 Years (234)	204 (87.2)	30 (12.8)	
**Occupation**	Farmer (221)	176 (79.6)	45 (20.4)	0.39
	Others (87)	73 (83.9)	14 (18.1)	

### Compliance rate versus behavioral and service related factors

Respondents were asked about their awareness of onchocerciasis, the responsible vector (insect) for the transmission of the disease, way of transmission and the sign and symptoms of the disease. The rate of compliance to treatment was higher (83.0%) among respondents who knew/heard about onchocerciasis compared to those who did not know about the disease (35.7%) (P < 0.05). Relatively higher rate of treatment compliance was reported by respondents who know the responsible vector, means of transmission and signs/symptoms of onchocerciasis (84.6%, 79.2% and 82.8% respectively) compared to their counter parts, but it was not statistically significant. Furthermore, the respondents were asked about the responsible body for distributing the tablet. Those who said CDDs, were significantly over represented (89.5%) under treatment compliance compared to those who said health extension workers (55.6%), health professionals (50.0%), and others (46.7%) (p < 0.05). On top of this, those respondents who know at least one CDD in their village had significantly higher rate of treatment compliance (89.2%) compared to those who do not know any CDD in their village (43.9) (p < 0.05) (Table 4).

**Table 4:** **Compliance rate versus behavioral factors among the study participants in Kabo area, southwestern Ethiopia, 2012**

**Variables (N = 308)**	**Category (no.)**	**Compliance with Ivermectin**		**P-value**
		**Yes (%)**	**No (%)**	
**Know (heard about) the disease onchocerciasis**	Yes (294)	244 (83.0)	50 (17.0)	0.00
	No (14)	5 (35.7)	9 (64.3)	
**Know the vector (Black fly) for transmission**	Yes (26)	22 (84.6)	4 (15.4)	0.61
	No (282)	227 (80.5)	55 (19.5)	
**Know mode of transmission (Black fly bite)**	Yes (24)	19 (79.2)	5 (20.8)	0.83
	No (284)	230 (81.0)	54 (19.0)	
**Know sing and symptoms (at least one)**	Yes (227)	188 (82.8)	39 (17.2)	0.82
	No (81)	61 (75.3)	20 (24.7)	
**The responsible body for distributing the drug**	CDDs (237)	212 (89.5)	25 (10.5)	0.00
	HEWs (36)	20 (55.6)	16 (44.4)	
	Health professionals (20)	10 (50.0)	10 (50.0)	
	Others (15)	7 (46.7)	8 (53.3)	
**Know any CDD in their village**	Yes (251)	224 (89.2)	27 (10.8)	0.01
	No (57)	25 (43.9)	32 (56.1)	
**Know how CDDs recruited**	Yes (121)	101 (83.5)	20 (16.5)	0.35
	No (168)	148 (79.1)	20 (20.9)	
**Attend village meeting on CDTI at least once**	Yes (174)	144 (82.8)	30 (17.2)	0.33
	No (134)	105 (78.4)	29 (21.6)	
**Onchocerciasis a serious disease**	Yes (260)	237(91.2)	23(8.8)	0.00
	No (33)	10(30.3)	23(69.7)	
**Onchocerciasis common in their village**	Yes (243)	225 (92.6)	18(7.4)	0.00
	No (47)	22 (46.8)	25 (53.2)	
**Risk perception to onchocerciasis**	Yes at risk (252)	222(88.1)	30(11.9)	0.00
	Not at risk (34)	16 (27.6)	18 (72.4)	
**Perceived performance of CDDs**	Good (217)	191(88.0)	26 (12.0)	0.00
	Poor (91)	58 (63.7)	33 (36.3)	
**Perceived importance of CDTI**	As very important (281)	246(81.7)	55 (18.3)	0.01
	As obligation (27)	3 (42.9)	4 (57.1)	
**Perception towards the program (can control onchocerciasis)**	Yes (274)	244(89.1)	30(10.9)	0.00
	No (3)	1(33.3)	2(66.7)	
**Know a person who stopped treatment**	Yes (16)	9(56.2)	7 (43.8)	0.01
	No (292)	240 (82.2)	52 (17.8)	
**Perceived reason of stopping the treatment**	Sever effect of the drug	2 (40.0)	3 (60.0)	0.53
	Lack of good case mgt.	5 (71.4)	2 (28.6)	
	Others	2 (50.0)	2 (50.0)	

The magnitude of compliance to treatment was significantly higher for respondents who perceive onchocerciasis as a serious disease (91.2%) and common in their village (92.6%) compared to those who perceive onchocerciasis as not serious (30.3%) and not common in their village (46.8%) (p < 0.05). Similarly, those who perceive that they are at risk of contracting the disease had significantly higher treatment compliance rate (97.4%) compared to those who perceive themselves not at risk of getting onchocerciasis (27.6%) (p < 0.05). In addition, respondents who perceived CDDs are performing their work well had significantly higher compliance rate (89.1%) compared to those who deem CDDs are poor in performance (12.1%) (p < 0.05). Furthermore, respondents who perceived that CDTI as very important (87.9%) and the program can control onchocerciasis (89.1%) had significantly higher compliance rate compared to those who look at CDTI as an obligation (7.4%) and those who believe the program can’t control onchocerciasis (33.3%) (p < 0.05) (Table 4).

None of the service-related factors (timing of service provision, decision maker of service periods, places of service provision and sources of information about the service) was associated with compliance to ivermectin treatment.

### Independent predictors of compliance with CDTI

Crude analysis of socio-demographic variables (Table 3) showed that age, educational status, ethnicity and length of stay in the study area were significantly associated with compliance to ivermectin treatment (p < 0.05). On the other hand; sex, marital status, religion, occupation, family size and monthly income did not show statistically significant association. While among the behavioral factors (Table 4); awareness of onchocerciasis, perception towards onchocerciasis, perceived performance of CDDs, perception towards the CDTI and the program, knowing a person stopped treatment in their village, awareness regarding a person (professional) responsible for distributing ivermectin and knowing at least one CDD in their village had shown significant association with compliance to ivermectin treatment. Knowledge on the responsible vector for the transmission, mode of transmission and sign and symptoms, previous family history of onchocerciasis, awareness on how CDDs recruited, awareness of other useful effects of ivermectin, methods of ivermectin dose determination, existence of problem with drug distribution, and participation in community meeting regarding CDTI from the behavioral factors did not show statistically significant association with compliance to CDTI in the bivariate analysis.

All variables that were significant in bivariate analysis were used in the multivariate logistic regression analysis to identify independent predictors of compliance to CDTI. Consequently, five variables were found to have statistically significant association with compliance to ivermectin treatment after adjusting for other variables. Age, length of stay, risk perception to onchocerciasis, perception towards the performance of CDDs and familiarity with at least one CDD in their village independently showed significant association (Table 5).

**Table 5:** **Independent predictors of CDTI in Kabo area, southwestern Ethiopia, 2012**

**Variables**		**Compliance with Iv.**		**Crude OR (95% CI)**	**Adjusted OR (95% CI)**
		***Yes***	***No***		
**Age group**	**≤35 Years**	160	53	Reference	Reference
	**>35 Years**	89	6	4.91 (2.03, 11.88)	5.48 (1.97, 15.23)
**Length of stay**	**<10 Years**	45	29	Reference	Reference
	**≥10 Years**	204	30	4.38 (2.40, 8.02 )	3.86 (1.83, 8.11)
**Perceived risk**	**Yes at risk**	222	30	8.33 (3.84, 18.05)	7.05 (2.70, 18.43)
	**Not at risk**	16	18	Reference	Reference
**Perceived performance of CDDs**	**Good**	191	26	4.18 (2.31, 7.56)	2.35 (1.15, 4.83)
	**Poor**	58	33	Reference	Reference
**Familiarity with CDDs (at least one)**	**Yes**	214	37	3.64 (1.92, 6.88)	2.83 (1.26, 6.40)
	**No**	35	22	Reference	Reference

Respondents with age greater than 35 years old were 5.48 times more likely to comply with CDTI compared to those whose age was less than or equal to 35 years (adjusted odds ratio = 5.48; 95% CI: 1.97 - 15.23). Similarly, individuals who stayed in the study area for more than or equal to ten years were 3.86 times more likely to comply with the treatment compared to those who stayed in the area for less than ten years (adjusted odds ratio = 3.86; 95% CI: 1.83 - 8.11). The respondents who perceived themselves to be at risk of onchocerciasis infection were 7.05 times more likely to comply with the treatment compared to those who did not (adjusted odds ratio = 7.05; 95% CI: 2.70 - 18.43). In addition, respondents who perceived that CDDs are doing their work well were 2.35 times more likely to comply with CDTI compared to their counter parts (adjusted odds ratio = 2.35, 95% CI: 1.15, 4.83). Moreover, respondents who know at least one CDD in their village were 2.83 times more likely to comply with CDTI compared to those who did not know any CDD in their village (adjusted odds ratio = 2.83; 95% CI: 1.26 - 6.40) (Table 5).

## Discussion

The results of this study indicated that 80.8% of the respondents took ivermectin during the 9^th^ round of the annual ivermectin distribution. Although the treatment coverage seems good, there is a need to attain and maintain a high coverage of CDTI; at least 90%, for elimination of onchocerciasis as a public health problem [17,25]. In this study, almost one fifth (19.2%) of eligible respondents in the community were non-compliers to CDTI. This implies that members of the community who do not take the treatment may serve as a reservoir for continued transmission of onchocerciasis in the area. The major reasons for not taking the annual treatment include: being in the field (farming) during the campaign day, homes not being visited by CDDs, not being informed about the campaign day, do not trust the treatment, and other reasoning’s such as fear of side effects. A study conducted in Uganda showed that among those individuals eligible for treatment, 17.4% were reported as non-compliers in the tenth round of ivermectin distribution [22], although their study did not indicate the reasons. Another study conducted in Nigeria reported that absenteeism during the campaign day was the major reason for missing the treatment [26].

The need to understand factors contributing to compliance to CDTI is paramount for improving the efforts in the prevention and control of onchocerciasis as an economic and public health problem. Findings of this study showed that age over 35 years, length of stay, perception about risk of contracting onchocerciasis, perception of the performance of CDDs and familiarity with CDDs in their villages were the factors found to be independent predictors of compliance with ivermectin treatment. Older age groups (age >35 years) had a positive association with CDTI compliance and were almost five and half times more likely to comply with the treatment compared to younger ones. The finding is consistent with a study conducted in Nigeria and Cameroon [19,27]. The reason may be attributed to the fact that younger individuals are relatively highly mobile and likely to travel outside the village for work and other opportunities or they could be reluctant and miss the annual treatment. Moreover, a shorter period of ivermectin distribution that lasts for just a few days possibly increases the number of non-compliers. Respondents who stayed in the study area for ten years and more were more likely to comply with the treatment compared to those who stay in the study area less than ten years. This might be due to the fact that the more a person stays in the endemic area, the more he/she acquires relevant information on the benefits of the treatment from the existing intervention package in the area.

The strongest factor associated with compliance to CDTI in this study was perceived personal risk of onchocerciasis. Individuals who perceive that they are at risk of getting the infection were nearly seven times more likely to comply with CDTI compared to those who consider themselves as free from risk of the infection. Other studies also showed that when people believe that personal susceptibility to onchocerciasis is high then, adherence to the treatment is also high [16,18,22,28].On top of this, the finding also demonstrated a pivotal role of CDDs in the success of CDTI. Perceived good performance of CDDs and knowing at least one CDD was associated with increased compliance. Thus, respondents who perceive CDDs are doing their work well were more likely to comply with the treatment and those who know at least one CDD in their village were likely to comply with CDTI compared to their counter parts. This finding is in congruent with the studies conducted in southwestern Ethiopia, Nigeria and Uganda [16,19,22]. The study conducted in southwestern Ethiopia (Bebeka coffee plantation) revealed insufficient knowledge on the side of CDDs resulted in hampered acceptability of CDDs performance by the community [16]. It is important that CDDs are able to carry out their tasks and need regular training [29].

One of the limitations of this study was that we used a cross-sectional study design, which is a snapshot of a single point in time and misses the seasonal trends of CDTI compliance rate in the study area. Since the documentation and registration system of CDTI implementation was weak in the area, to avoid recall bias we conducted a cross-sectional study just three weeks after the annual ivermectin distribution. The other limitation was that a relatively higher degree of accuracy (5% degree of precision) was used which limits our sample size to 308.

## Conclusion

In conclusion, the annual treatment coverage of ivermectin in the study area seems good, but this does not mean that all eligible individuals have been complying with the treatment. There are members of the community that do not comply with the annual treatment particularly younger individuals, recent comers and reluctant. Hence, a special emphasis needs to be given for these segments of population to increase awareness about perceived personal risk of onchocerciasis and so to address the issue of non-compliance. Moreover CDDs need to be continuously motivated and supported to perform their work well and also they should have to develop a rapport with the community members. On top of this, there is a need for further operational research for achieving and sustaining high level of coverage.
